# How Climate Change Beliefs among U.S. Teachers Do and Do Not Translate to Students

**DOI:** 10.1371/journal.pone.0161462

**Published:** 2016-09-07

**Authors:** Kathryn T. Stevenson, M. Nils Peterson, Amy Bradshaw

**Affiliations:** 1 Fisheries, Wildlife, and Conservation Biology Program, Department of Forestry & Environmental Resources, North Carolina State University, Raleigh, North Carolina, United States of America; 2 Department of Zoology, NC State University, Raleigh, North Carolina, United States of America; TNO, NETHERLANDS

## Abstract

Research suggests climate change beliefs among science teachers mirror those of the general public, raising questions of whether teachers may be perpetuating polarization of public opinion through their classrooms. We began answering these questions with a survey of middle school science teachers (n = 24) and their students (n = 369) in North Carolina, USA. Similar to previous studies, we found that though nearly all (92.1%) of students had teachers who believe that global warming is happening, few (12%) are in classrooms with teachers who recognize that global warming is anthropogenic. We found that teacher beliefs that global warming is happening and student climate change knowledge were the strongest predictors of student belief that global warming is happening and human caused. Conversely, teacher beliefs about human causes of global warming had no relationship with student beliefs, suggesting that science teachers’ low recognition of the causes of global warming is not necessarily problematic in terms of student outcomes. These findings may be explained by previous research suggesting adolescents interpret scientific information relatively independently of ideological constraints. Though teacher polarization may be problematic in its own right, it appears that as long as climate change information is presented in classrooms, students deduce anthropogenic causes.

## Introduction

Although nearly all (>95%) climate scientists attribute global warming to human activities[1], only about half of U.S. adults agree[2,3], and similar polarization can be found across many parts of the world [4,5]. Researchers attribute this persistent disconnect between scientific consensus and public perceptions to individuals’ heavy reliance on worldview and political ideology, which drive individuals to seek information from ideologically compatible sources [6] and shape their interpretation of new information [7]. Although worldview and political ideology are the overwhelming drivers of climate change perceptions [8], some research does suggest that climate-specific knowledge can predict acceptance of anthropogenic global warming as well as climate change concern among adolescents [9] and adults [10,11], indicating that climate education may have the potential to overcome ideologically-driven polarization. However, a recent survey of U.S. science teachers suggests that climate literacy is low and polarization is high among the very people who have the potential to leverage education to unite public opinion [12].

Gaps between teachers’ knowledge about climate science and the scientific consensus of anthropogenic global warming are concerning when considering the importance of climate change education in schools. A 2016 national survey of US teachers found that although a vast majority of middle and high school science teachers (70% and 87%, respectively), dedicate at least an hour of instruction to climate change, 30% emphasize that global warming is due to natural causes and 31% teach “both sides” (i.e., that recent global warming is due to human activity but that many scientists think it is due to natural causes)[12]. Similarly, less than half of teachers (30% of middle school science teachers and 45% of science teachers) responded to the correct proportion of climate scientists who think global warming is caused mostly by human activities [12]. The disconcerting fact that so little time is dedicated to climate change (median of 1–2 hours [12]) aside, these numbers call into question the quality of climate education our children are receiving. Perhaps the most troubling aspect of this study was that teachers’ political ideology was the most powerful predictor of their classroom approach [12]. This would suggest that the biggest determinant of how our children are presented information about climate change in science classrooms is driven by a factor that is incredibly hard to change and largely independent from science [13].

Despite the role that politics seems to play in how climate change is addressed in science classrooms, research on how students learn about climate change is more encouraging. Among adults, worldview and political ideology seem to be primary drivers of climate change beliefs [8], and education level and scientific literacy may further entrench people into their ideologically-based beliefs [6,14]. Among adolescents, however, worldview is associated with polarized climate change beliefs at low levels of climate change understanding, but climate education appears to eliminate ideological differences once adolescents understand key scientific concepts associated with climate change [9]. Although changing climate change perceptions among adults may be difficult because of the strong influence of worldviews and political ideology [8], educational interventions may be effective among adolescents at fostering climate change knowledge, belief in anthropogenic global warming, behavioral intentions and reported behavior around climate change [15,16]. This research points to the possibility that although teacher beliefs may influence how climate change is presented in the classroom [12], younger audiences may be able to better separate scientific evidence from its politically driven context.

Although climate change knowledge may more directly influence beliefs among adolescents than among adults, other insights on adolescent perceptions of climate change suggest that socio-cultural factors such as gender, ethnicity, and descriptive norms may shape climate change beliefs, raising questions about the relative importance of teacher climate change beliefs on those of their students. Several studies have shown gender effects around adolescent perception of climate change, with girls more concerned about climate change than boys [9]. These mirror similar studies among adults [17,18] and suggest that gender-related social norms may influence how young children relate to climate change. Similarly consistent with studies among adults [18], non-White adolescents were also found to have higher levels of acceptance of anthropogenic global warming than Whites [9]. Among adults, the explanation for this difference may be rooted in environmental justice issues, with non-White populations disproportionally exposed to the negative effects of climate change [19]. It is possible, even likely, that children adopt the perspectives of their parents or even recognize their own vulnerability to climate change. Other studies suggest that descriptive norms may play an important role in forming climate change beliefs. In one study, perceived skepticism among parents and peers were the most important predictors of climate change skepticism among adolescents, over political and value orientations, subjective knowledge, and self-efficacy [20]. In another, teens whose parents had high climate change risk perceptions were more likely to seek information about climate change [21], suggesting that parent beliefs drive behaviors among their children. Evidence for these socio-cultural drivers of climate change beliefs along with evidence that teachers can spur climate change engagement [22] suggest that just as parents and peer beliefs influence adolescent climate change perceptions, teacher climate change beliefs may also the climate change beliefs of their students.

In this study we tested four hypotheses about how teachers’ beliefs regarding climate change influence the beliefs of their students with a case study in North Carolina, USA: 1–2) teacher belief that climate change is happening would positively impact student belief that climate change is happening and human caused, and 3–4) teacher belief that climate change is human caused would positively impact student belief that climate change is happening and human caused. We used middle school subjects in our case study since middle school students are developmentally able to grasp complex issues such as climate change [23], seem less susceptible to worldview-driven biases around climate change [9], but are influenced by socio-cultural factors such as gender and ethnicity [9], parents, peers, and teachers [21,24,25]. Additionally, we focused on coastal North Carolina counties for our study since people in coastal areas are more apt to perceive threats of climate change since they will likely be most directly affected by climate change impacts such as sea level rise and loss of coastline [26]. Further, as we are interested in how politically-driven climate change beliefs of teachers may impact beliefs of students, we chose North Carolina because of the highly politicized context surrounding discourse related to sea level rise, climate change, and climate education [27]. To promote face validity in the study we also control for known drivers of belief in anthropogenic climate change: gender, climate knowledge, and ethnicity.

## Methods

### Ethics Statement

The North Carolina State University institutional review board (IRB # 2961) approved this study. All participants provided written informed consent. Students and their parents/guardians were given either a Passive Consent form or an Active Consent form, per the preference of the teacher and/or school. The Passive Consent form was only signed and returned if the parents/students did not want to participate. The Active Consent form was signed and returned to indicate consent to participate in the study.

### Sampling

For this study we chose to focus on middle school students because they are at an age where they start applying their knowledge to real-world situations and making informed decisions [28]. We sampled in two stages—teachers and students. First, we compiled a list of all middle school science teachers in the 20 coastal North Carolina counties by visiting the school websites, assembling faculty rosters, and contacting the schools to authenticate the faculty rosters. We randomly selected 150 of the 353 teachers for the study. However, 27 of the 150 selected teachers were removed from the study because two of the school districts did not permit teachers to participate. Of the 123 remaining teachers selected for the study, 36 responded and 24 agreed to participate. The participating teachers were each asked to randomly select a class to be involved in the study by flipping a coin. From March through May of 2013, we visited the 24 classrooms and surveyed each student in person. This sample consisted of 90 sixth graders, 95 seventh graders, and 182 eighth graders—yielding a total of 369 students. Most students were in the age range of 11 to 14 years old, but 17 of the students were 15 years old. A majority (81.8%) of the students completed the entire survey. Most of the students in the sample were female (54.8%) and White (60.5%), with fewer students identifying as African American (15.6%), Hispanic (7.3%), American Indian (1.3%), Asian (1.0%), multi-racial (10.9%), and other (3.4%).

### Instrument development

To measure student and teacher beliefs in AGW, we used two items from one of the only published instruments used with both adult and adolescent populations [29]. One item measured whether students and teachers thought GW was happening and another measured whether they thought it was human caused (Table 1). We measured climate change knowledge among students with a modified version of the climate knowledge scale in Tobler et al. [30], which addresses climate science, causes, and impacts (Table 2). We pretested this instrument with 92 middle school students as outlined in Stevenson et al. [9]. We asked students to give written and verbal feedback when taking the instrument and conducted nine cognitive interviews [31] to elicit further general feedback on question wording and test alternative wording of questions when appropriate.

**Table 1:** Items measuring belief in global warming and human causes.

Question	Student Mean	Student SD	Teacher Mean	Teacher SD
Recently you may have noticed that global warming has been getting some attention in the news. Global warming refers to the idea that the world’s average temperature has been increasing over the past 150 years, may be increasing more in the future, and that the world’s climate may change as a result.				
What do you think? Do you think that global warming is happening?				
Yes…	6.94	1.93	1.88	1.73
a)…and I'm extremely sure (9)				
b)…and I'm very sure (8)				
c)…and I'm somewhat sure (7)				
d)…but I'm not at all sure (6)				
No…				
e)…and I'm extremely sure (1)				
f)…and I'm very sure (2)				
g)…and I'm somewhat sure (3)				
h)…but I'm not at all sure (4)				
Or…				
i) I don't know (5)				
Assuming global warming is happening, do you think it is…	2.99	0.93	3.13	0.34
a) Caused mostly by human activities (4)				
b) Caused by both human activates and natural changes in the environment (3)				
c) Caused mostly by natural changes in the environment (2)				
d) None of the above because global warming isn't happening (1)				
e) Other*				

*We excluded respondents who selected this choice from final analysis (n = 9).

Numbers beside each answer choice represent coding used (N = 369).

**Table 2:** Climate change knowledge scale.

Topic	Item wording	% Correct	SD
**Climate Change Science**	Burning oil, among other things, produces carbon dioxide (CO_2_)	81.6	0.39
	Carbon dioxide (CO_2_) is a greenhouse gas.	72.1	0.45
	Greenhouse gasses partly keep the Earth’s heat from escaping into space.	55.3	0.50
	Carbon dioxide (CO_2_) is harmful to plants	72.6	0.45
	The ozone hole is the main cause of the greenhouse effect.	49.6	0.50
	At the same quantity, carbon dioxide (CO_2_) is more harmful to the climate than methane.	64.0	0.48
**Climate Change Causes**	The global carbon dioxide (CO_2_) concentration in the atmosphere has increased during the past 250 years.	80.2	0.40
	The increase of greenhouse gasses is mainly caused by human activities	77.5	0.42
	With a high probability, the increase of carbon dioxide (CO_2_) is the main cause of climate change.	55.3	0.50
	Climate change is mainly caused by natural variations (such as changes in solar radiation and volcanic eruptions)	50.9	0.50
	The last century’s global increase in temperature was the largest during the past 1000 years.	63.1	0.48
	The decade from 2000 to 2009 was warmer than any other decade since 1850.	69.9	0.46
	The amount of (CO_2_) in the atmosphere has reached the same levels within the past 650,000 years.	68.0	0.47
**Change Impacts Climate**	**For the next few decades, the majority of climate scientists expect…**		
	… an increase in extreme events, such as droughts, floods, and storms	77.8	0.42
	… a warmer climate to increase the melting of polar ice, which will lead to an overall rise of the sea level.	79.4	0.40
	… a cooling-down of the climate	74.8	0.43
	… a warmer climate to increase water evaporation, which will lead to an overall decrease of the sea level.	63.1	0.48
	… the climate to change evenly all over the world.	67.2	0.47
	… a precipitation increase in every region worldwide.	53.9	0.50

We drew these questions from Tobler et al. [30] and modified questions based on pretesting with middle school students (*n* = 92).

Percentage correct represents the percentage of respondents whose answers reflect current scientific understanding (n = 369).

### Data Analysis

Using STATA version 14.1, we used multiple linear regression to model student beliefs that GW is happening and human caused as a function of teacher belief that GW is happening and teacher belief that GW is human caused. We also controlled for student climate change knowledge as predictors and controlled for student gender and race. We compared both ethnicity and race in our sample to student population data available through the NC Department of Public Instruction. Because we found our sample underrepresented males (45.2% in our sample versus 51.0% in coastal NC; t = 2.85, p = 0.005), we weighted our sample to adjust for this difference.

## Results

Most students in our sample had teachers who believed that global warming is happening (92.1%) and caused by a mixture of natural and human causes (88.0%). Thus only 12% of students were associated with teachers whose views on climate change causes matched the scientific consensus that global warming is caused mostly by human activities (Table 1) [32]. Smaller percentages of students thought that global warming was happening (81.8%), but a greater percentage (30.2%) thought that climate change is mostly caused by humans. Students scored an average of 12.8 out of a maximum of 19 on the climate change knowledge scale (SD = 2.73). For mean values for each question in the climate knowledge scale please see Tables 1 and 2.

**Fig 1:**
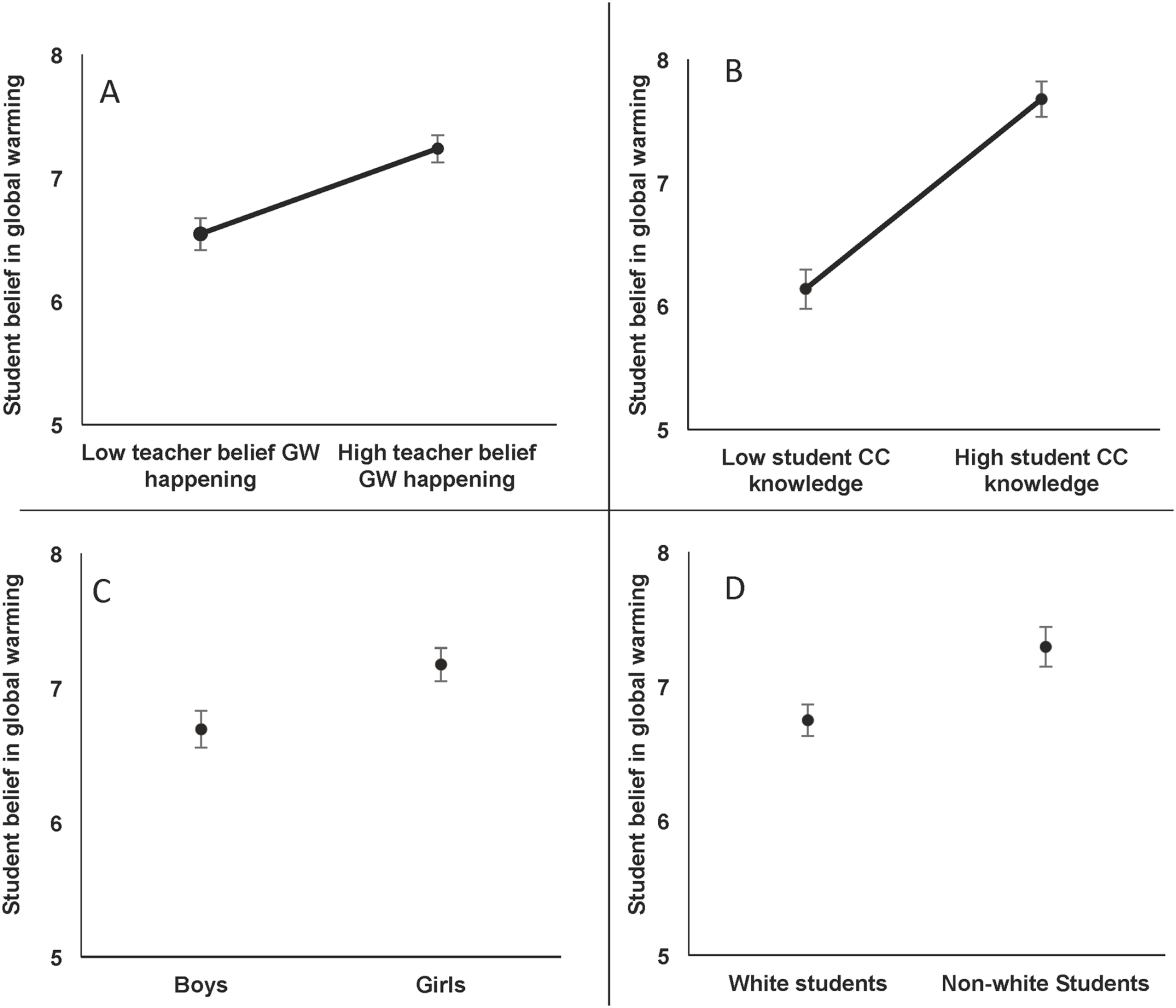
Significant predictors of student belief in global warming (n = 369). Student belief scale ranged from 1 (strong disbelief in GW) to 9 (strong belief in GW). Predicted values were derived from model 1 in Table 3. Low and high values of teacher belief in GW and student CC knowledge reflect the predictions for the 10^th^ and 90^th^ percentiles, respectively. Accordingly, larger differences between two values indicate larger effects sizes, with all other variables held constant. Error bars represent 95% confidence intervals.

**Fig 2:**
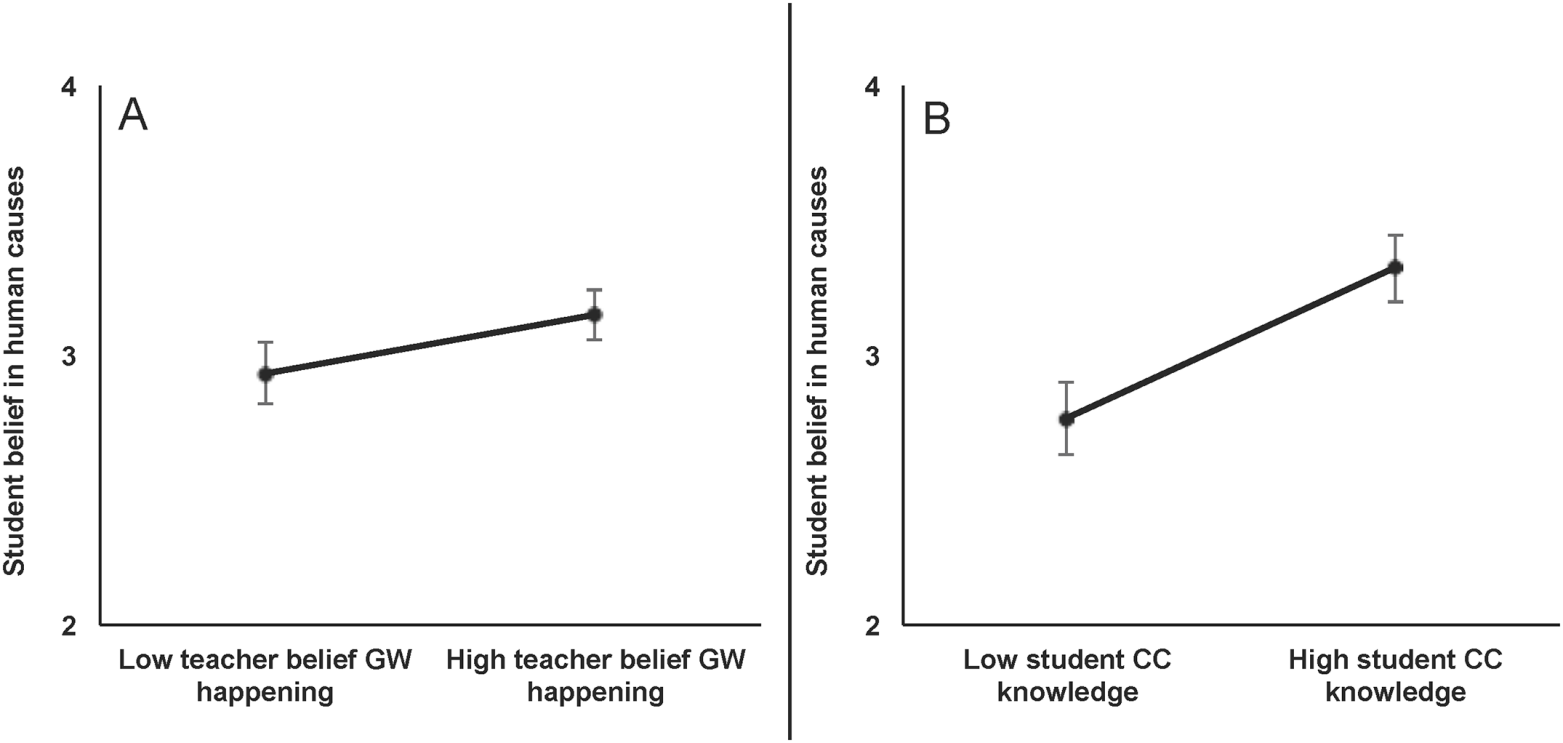
Significant predictors of student belief in human causes of global warming (n = 369). Student belief scale included 1 (nothing causes global warming because it is not happening), 2 (belief in human causes), 3 (belief in human and natural causes), and 4 (belief in human causes over natural causes). Predicted values were derived from model 2 in Table 3. Low and high values of teacher belief in GW and student CC knowledge reflect the predictions for the 10^th^ and 90^th^ percentiles, respectively. Accordingly, larger differences between two values indicate larger effects sizes, with all other variables held constant. Error bars represent 95% confidence intervals.

We found that only teacher belief that climate change was happening predicted the beliefs of their students. Specifically, we found that teachers’ belief that global warming was happening was a strong, positive predictor of students’ belief that global warming was happening and human caused (Table 3, Figs 1 and 2). However, teacher belief that global warming was human caused had no relationship with student belief that global warming was happening or human caused (Table 3). We also found that student climate change knowledge was a strong, positive predictor of students’ belief that global warming is happening and human caused, supporting our assertion that if students can assemble the basic background information they will figure out human causes independent of teachers’ belief in them (Table 3, Figs 1 and 2). Also, we found that non-White and female students were more likely to believe global warming was happening (Table 3, Fig 1).

**Table 3:** Regression Models Predicting Student Belief that Global Warming is Happening and Global Warming is Anthropogenic.

	Model 1: Student belief that GW is happening			Model 2: Student belief that GW is human caused		
Item	Beta	Std Beta	p	Beta	Std Beta	p
Teacher Belief GW is Happening	0.24	0.23	0.002	0.07	0.17	0.008
Teacher Belief GW is Human caused	-0.08	-0.01	0.80	0.09	0.04	0.46
Student Climate Change Knowledge	0.23	0.32	<0.001	0.08	0.27	<0.001
Student Gender*	0.48	0.12	0.015	-0.02	-0.01	0.81
Student Race: Non-White	0.58	0.14	0.004	0.16	0.06	0.10

*Gender: 0 = male, 1 = female

R^2^ for Model 1 = 0.16

R^2^ for Model 2 = 0.10

N = 369

Because several other studies have found correlations between belief that climate change is happening and human caused [33,34], we explored the possibility of collinearity between teacher beliefs. We found these variables significantly, but weakly correlated (r = 0.24, p <0.001). We ran the same regression models reported in this study with the teacher belief that global warming is happening variable omitted and teacher belief in human causes for climate change remained a non-significant variable in models predicting both student belief that climate change was happening and student belief that climate change had human causes.

## Discussion

Our findings suggest convincing teachers that climate change is real, but not necessarily human caused, may have profound impacts on students. Research suggests that individuals are more receptive to climate change communication from trusted messengers [35], and our results support findings suggesting that teachers are among the trusted messengers for adolescents [25]. Most teachers (98% in the national survey [12] and 92% in our sample) believe that global warming is happening, and our results suggest that those beliefs may transfer to their students, with strength in agreement that global warming is happening positively correlated between teachers and students. Teachers are more polarized on the causes of global warming [12], but encouragingly, our results suggest that these beliefs do not transfer to students. Overwhelmingly, research suggests that teachers’ disagreement with the scientific consensus about climate change causes is likely rooted in political ideology and worldviews [8]. However, research among adolescents suggests that these factors may not shape climate change beliefs among children to the same degree as they do among adults [9]. It is possible that if teachers are effective messengers of the reality of climate change, students are able to distill its anthropogenic causes even when teachers do not believe in anthropogenic causes. Although our results suggest that teacher beliefs about human causes have little relationship with student beliefs, we did not examine what actually occurs in the classroom. Future research should address whether these trends hold among teachers who send “mixed messages” that many scientists believe that climate change is human-caused while others attribute to natural causes, as was reported in a national science teacher survey [12].

Teachers’ beliefs that global warming was happening mattered nearly as much as whether students had knowledge about climate change in terms of predicting students’ beliefs in climate change (Table 1, Fig 2). Although adolescents seem able to separate climate change knowledge from ideological biases [9], research has shown that their climate change beliefs are impacted by factors such as the perceived beliefs of their friends and family [20], and their information-seeking behavior is influenced by the beliefs of their parents [21]. This influence of teachers on adolescents’ belief may relate to issue saliency. Children whose families discuss climate change were more likely to seek information, regardless of how concerned parents were about climate change, suggesting that engagement stemming from discussion may boost issue saliency for adolescents [21]. Applied in a classroom context, it seems reasonable that belief in climate change existing among teachers operates as a pre-requisite for discussing the subject, certainly more so than considering it a human caused phenomenon, and that such discussion boosts issue saliency for students.

Beyond the role of teacher beliefs, our findings support the growing literature suggesting minorities and women present more belief in and concern about climate change than their counterparts. Several studies have documented similar differences [9,24], and future research should address the role of gender socialization, racial and ethnic identity, and exposure to climate change risks as potential factors shaping climate change perceptions among adolescents. In particular, research should investigate how these dynamics may compare to those among adults, and whether these demographic-related influences persist from adolescence into adulthood. Similarly, future research should explore how dynamics of teacher beliefs about climate change shape the way teachers engage their students pedagogically. Our results suggest that there is likely a nuanced relationship between how teacher beliefs influence student beliefs around climate change. We agree with many of the implications from the survey of national science teachers, namely that teachers need professional development that focuses on communicating the consensus of anthropogenic global warming, improves teachers’ understanding of climate science, and attends to teachers’ political ideologies and worldviews [12]. However, we suggest that future research work to better understand how teacher beliefs may influence specific aspects of classroom instruction because teacher belief in human caused climate change appears to have no measurable impact on key student outcomes.
